# Toothache Drops

**Published:** 1868-02

**Authors:** 


*Toothache Drops.*—An admirable mixture is made by combining in equal parts, creasote, laudanum, chloroform, tinct. aconite, tinct. of iodine, and lead water. To be applied on a pellet of cotton.

